# Root contraction does not increase long-term population growth in *Pediocactus bradyi*, an endangered cactus of Northern Arizona

**DOI:** 10.1093/aobpla/plaf074

**Published:** 2025-12-30

**Authors:** Julieta Rojas-Pimentel, Eugenio Larios, Edgar J González

**Affiliations:** Departamento de Ecología y Recursos Naturales, Facultad de Ciencias, Universidad Nacional Autónoma de México, Circuito Exterior s/n, Mexico City 04510, Mexico; Programa Educativo Licenciatura en Ecología, Universidad Estatal de Sonora, Ley Federal del Trabajo s/n, Apolo, Hermosillo 83100, Sonora, Mexico; Ecología para la Conservación del Gran Desierto, A.C., Roberto Romero 63, Jesús García, Hermosillo 83140, Sonora, Mexico; Departamento de Ecología y Recursos Naturales, Facultad de Ciencias, Universidad Nacional Autónoma de México, Circuito Exterior s/n, Mexico City 04510, Mexico; Populations & Communities

**Keywords:** globose cactus, integral projection model, perturbation analysis, population dynamics, root elongation, root shrinkage

## Abstract

*Pediocactus bradyi*, a semi-globose cactus endemic to northern Arizona, displays a root-contraction mechanism to survive extreme drought: its roots contract, pulling the stem below ground during dry periods, re-emerging once rains return. To quantify how root contraction shapes population dynamics, we developed an integral projection model based on 31 years of demographic data from a *P. bradyi* population on the Navajo Nation Off-Reservation Trust Land. We explored two scenarios: one including root contraction and one excluding it. We found that, being ∼10% of the individuals and mostly confined to smaller individuals, root contraction did not have an effect on the long-term population growth [λ = 1.041 (1.039, 1.303) with contraction vs. λ = 1.044 (1.035, 1.289) without]. Also, we show that larger individuals have higher survival and reproductive rates, while growth declines beyond 35 mm in diameter. An elasticity analysis confirmed that survival and growth are the main vital rates affecting population growth, followed by root elongation after contraction. Thus, while root contraction may improve individual survival, elongation is in fact more important at the population level. Therefore, as with most cacti species, conservation efforts should focus on ensuring the survival of large individuals irrespective of their root contraction status.

## Teaser

This study assesses whether root contraction affects long-term population dynamics of the endangered cactus Pediocactus bradyi in northern Arizona. Using 31 years of demographic data, models with and without root contraction yielded nearly identical population growth rates. Root contraction occurs infrequently, mainly in small individuals, and has negligible demographic impact. Population dynamics are driven primarily by survival and growth of large individuals, with growth slowing beyond a size threshold. Conservation should prioritize maintaining large individuals rather than root contraction status.

## Introduction

Plant functional traits, those individual characteristics that enhance population growth rate, are thought to evolve in relation to the environmental stress that plant species face in a given habitat and help them to persist (Grime 1977, Westoby et al. 2002, Adler et al. 2014, Reich 2014). Within this framework, Cactaceae provide a clear test case: chronic water limitation, high irradiance, and thermal extremes favour trait syndromes that buffer the population growth rate under aridity, which helps explain their peak diversity in deserts and xerophytic shrublands (Gibson and Nobel 1986, Goettsch et al. 2015, Hultine et al. 2023). These trait syndomes include unique morpho-anatomical and physiological adaptations, such as succulent and expandable stems that store water; spines and trichomes that regulate the incidence of solar radiation (Nobel 1978, Gibson and Nobel 1986, Darling 1989, Cornejo et al. 2003); a tendency to not present leaves (Bravo-Hollis and Sánchez-Mejorada 1978); thin, horizontal and generally shallow roots that allow the rapid absorption of water after prolonged drought (Cornejo et al. 2003); a crassulacean acid metabolism (CAM; Ting 1985); and a lower ratio of photosynthetic tissue compared to storage tissue (Hultine et al. 2016). One of the least studied morphophysiological strategies is root contraction, which allows cacti to survive under high drought conditions (Garrett et al. 2010).

Root contraction is a phenomenon in which roots reduce their length and in cacti, it allows them to protect from very high temperatures in rocky soils. It occurs because the xylem generates compressible networks that decrease the length of the roots and this mechanism pulls the plant into the soil (North et al. 2008, Garrett et al. 2010). Suberization in roots reduces the permeability of cell walls to water, affecting hydraulic conductivity (North and Nobel 1991). However, in contractile roots, a higher electrical conductivity has been observed, which implies a greater volume of water flowing per unit time (Steudle 2000, Zhang et al. 2019). This strategy is not only useful to reduce the contact surface that is under direct solar radiation; it allows them to transpire less water as they are at a lower temperature below the soil; having a higher hydraulic conductivity in their roots, allows them a greater water uptake; finally, according to Garrett et al. (2010), by being retracted, plants are less likely to be detected by herbivores.

Although there is a great deal of studies on the demography of the Cactaceae family (reviewed in Drezner and Lazarus 2008, Jiménez-Guzmán et al. 2024), studies that address the demographic consequences of particular traits, that could be hypothesized as adaptations to arid environments, are still relatively few. Serotiny, for instance—the retention of seeds within the cactus structures for more than a year—increases fitness in the face of environmental uncertainty in *Mammillaria pectinifera* (Peters et al. 2011). Seeds produced by this cactus are not released immediately after fertilization and can be stored within the ribs of the plant for years, helping to maximize fitness in the long term by reducing risk in variable environments, a phenomenon termed bet hedging in evolutionary theory. Clonality in *Opuntia rastrera* helps establish its populations in dry habitats, while sexually reproducing individuals recruit mostly in grassland habitats (Mandujano et al. 2001); these differences in life-history traits are reinforced by different selective pressures acting in these two different habitats. Finally, seed dormancy in the family Cactaceae is also a trait that, by increasing the relative contribution of seed survival, increases population growth rate through the formation of transient seed banks (Arroyo-Cosultchi et al. 2022).

*Pediocactus bradyi* is a semi-globose cactus species that contracts its roots during the extreme drought conditions to which it is subjected in certain years. According to Hughes (2005), when the species was monitored during periods of severe drought, many retracted individuals were found and later, under better humidity conditions, many of the retracted individuals came to the surface, 7 years after contraction. In this study, we analyse the population dynamics of *P. bradyi*, and explore the role root contraction may play in its population by modelling these dynamics under two scenarios: one with root contraction and another without it. We asked the following question: does root contraction increase population growth rate? We used data from three plots, surveyed over 31 years, and built an integral projection model to describe the population dynamics and compared both scenarios.

## Materials and methods

### Study species

*Pediocactus bradyi* (L. D. Benson), commonly known as Brady's pincushion cactus, is a polycarpic semi-globose cactus that flowers in March and April (LLIFLE 2020) and grows from 100 to 1200 m a.s.l. Its seeds are dispersed by animals of the order Rodentia (Hughes 1987). It has contractile roots, which allow it to retract itself below ground during periods of drought, and during the rainy season it emerges from the ground again (Hughes 1987, Roth 2008). The most frequent natural deaths are caused by rodents, insects, drought, old age, etc. (Hughes 1987). Several studies have indicated a decline in plant numbers attributed mainly to off-road vehicles disturbing their habitat, trampling and grazing by livestock, predation, and the incidence of uranium mines (Hughes 1987, Roth 2008, Hughes 2005). Their populations are denser in grazing plots (Hughes 1987). This species was declared threatened on 26 October 1979; according to Butterworth and Porter (2017), its populations were at <2500 individuals in 2017. It is very vulnerable due to its restricted distribution and small populations; however, it has been quite resilient due to its ability to retract (Hughes 1987).

### Study site

*Pediocactus bradyi* has a narrow distribution in the Arizona desert, specifically confined to the Navajo Nation Off-Reservation Trust Land, a reservation located in Coconino County, Arizona, USA, along the Colorado River and bordering Marble Canyon (Hughes 2005). Almost the entire region is a series of plateaus, more or less flat, with an approximate elevation of 1636 m a.s.l. There are high mountains of >3330 m and deep canyons. It is the largest Native American reservation in the USA with 62 362 km² of area. Almost half of the territory (with low altitudes) has a dry and hot climate in summer, but in the higher altitude plateaus it rains up to 30.5 cm per year, and forests of pine, oak, aspen, and spruce can be found. The desert and steppe territory is characterized by sub-zero temperatures in the winter, strong winds, sandstorms in the spring, and high evaporation. Precipitation occurs in two seasons: between January and March, there is snowfall, and between July and September, there are sporadic rains, sometimes with a magnitude that produces heavy floods (Whitehair et al. 2024); in the arid fore-summer, it rains almost nothing. The dominant vegetation along the canyon is composed of shrubs and grasses, the latter consisting of species such as *Hilaria jamesii*, *Bouteloua eriopoda*, *B. gracilis*, *Sporobolus cryptandrus*, and *Oryzopsis hymeniodies*. Shrubs are *Atriplex confertifolia* and *A. canescens* (Hughes 2005). The northwestern fringe of the Arizona desert is home to two threatened cactus species: *P. sileri* and *P. bradyi* (Hughes 1987). *Pediocactus bradyi* grows along the Cathedral Wash east of the Colorado River and south of the Sheep Springs Wash. The area lies between 35° and 37° N and between 106° and 112° W. In this area, three study plots were set: Badger, Soap Creek, and North Canyon; their precise locations are not revealed here due to the vulnerability status of the species.

### Database

The database covers the period from 1988 to 2019, and consists of the monitoring of 965 individuals. The database was provided by the US Geological Survey, which includes the label of each cactus sampled, its length (mm), the plot to which it belongs, the year in which the data were taken, as well as reproductive data, survival data, and whether or not they presented root contraction. No shrunk cactus was considered dead until it had remained in that state for >5 years; however, Hughes (2005) reports an individual who was shrunk for 7 years and eventually elongated.

### Vital rates modelling

For each vital rate (survival, growth, probability of reproduction, fecundity, root contraction, and root elongation), we built a full GLMM and a full GAMM (see Table 1 for details) with size as explanatory variable, and performed backward stepwise regression on the random effects of individual, year, and plot. The best model among the two simplified models was selected based on the Akaike selection criterion (AIC). We used the gamm4 library (Wood and Scheipl 2025) in R (R Core Team 2026) to perform the regression models.

**Table 1:** Structure of the models associated with each vital rate of the *Pediocactus bradyi* population studied.

Vital rate	Nomenclature	Link function	Probability distribution
Survival	*s*	logit	Binomial
Growth in non-retracted individuals	*G_u_*	identity	Gaussian
Growth in retracted individuals	*G_r_*	identity	Gaussian
Reproduction probability	*p_b_*	logit	Binomial
Number of reproductive structures	*b*	log	Poisson
Root contraction	*r*	logit	Binomial
Elongation	*u*	logit	Binomial
Size of offspring	*c*	identity	Density

To obtain the probability distribution of the offspring sizes, we used a clustering method (*K*-means, *K* = 2) to identify the recruits from among the individuals entering the population each year. This method fitted *K* normal distributions to the observed individual size distribution; we considered the normal distribution with the smallest mean as the offspring size distribution (*c* in Table 1).

In modelling the lifecycle of *P. bradyi*, we assumed that there is no mortality in retracted individuals, due to the infrequency of the phenomenon (two cases in 31 years). We also assume that elongation occurs at time *t*. We use the term ‘root elongation’ as the lengthening of the root after its contraction.

### Dynamic population modelling

With the vital rate models that best fit the data, we developed a four-summand annual IPM:


$$\begin{array}{c} n_{t+1}(z^{\prime })=\int K_{ur}(z^{\prime },z^{\circ })\cdot n_{r}(z^{\circ })dz^{\circ }+\int K_{uu}(z^{\prime },z)\cdot n_{u}(z)dz\;+\\ \;\;\int K_{ru}(z^{\circ },z)\cdot n_{u}(z)dz+\int K_{rr}(z^{\circ },z^{\circ })\cdot n_{r}(z^{\circ })dz^{\circ }, \end{array}$$

with


$$\begin{array}{c} K_{ur}(z^{\prime },z{\;}^{\circ })\;\;=\;u(z{\;}^{\circ })\times s(z{\;}^{\circ })\times G_{u}(z^{\prime },z{\;}^{\circ }),\\ K_{uu}(z^{\prime },z)\;\;=\;[1\text{–}r(z)]\times [s(z)\times G_{u}(z^{\prime },z)\;+p_{b}\times b\times p_{r}\times c(z^{\prime })],\\ K_{ru}(z{\;}^{\circ },z)\;\;=\;r(z),\;\mathrm{and}\\ K_{rr}(z{\;}^{\circ },z{\;}^{\circ })\;\;=\;1\text{–}u(z{\;}^{\circ }), \end{array}$$

where *n_t+_*_1_ represents the population structure of the size of individuals at time *t* + 1, *n_r_* the population structure of retracted individuals, *n_u_* the population structure of the non-retracted individuals, *K_ur_* is the kernel associated with the dynamics of retracted organisms at time *t* that are no longer retracted at time *t* + 1, *K_uu_* is the kernel associated with the non-retracted organisms that remain non-retracted, *p_r_* is the probability that a reproductive structure becomes a recruit, *K_ru_* is the kernel associated with the dynamics of the non-retracted organisms at time *t* that become retracted at time *t* + 1, *K_rr_* is the kernel associated with the dynamics of the retracted organisms at time *t* that continue at time *t* + 1, *z′* the size of individuals reached upon elongation at time *t* + 1, *z′* the size of individuals reached upon elongation at time *t*, and *z*° the size of the individuals before retracting. The rest of the functions are described in Table 1. *p_r_* was estimated as the ratio of the number of recruits by the number of reproductive structures observed in successive years.

### Population dynamics analysis

From the IPM, we extracted different metrics: the asymptotic population growth rate (λ), the stable size structure (*n*), the reproductive values, and the elasticity of λ to changes in the elements of *K_uu_*, *K_ur_*, *K_ru_*, and *K_rr_*. We also extracted asymptotic annual λ values by including the random effects associated with each year for both population dynamics (with and without root contraction).

The asymptotic λ and asymptotic annual λ values were obtained through iteration. Subsequently, we extracted the sensitivity and elasticity values for every vital rate as described by Griffith (2017).

## Results

### Vital rate analysis

**Figure 1:**
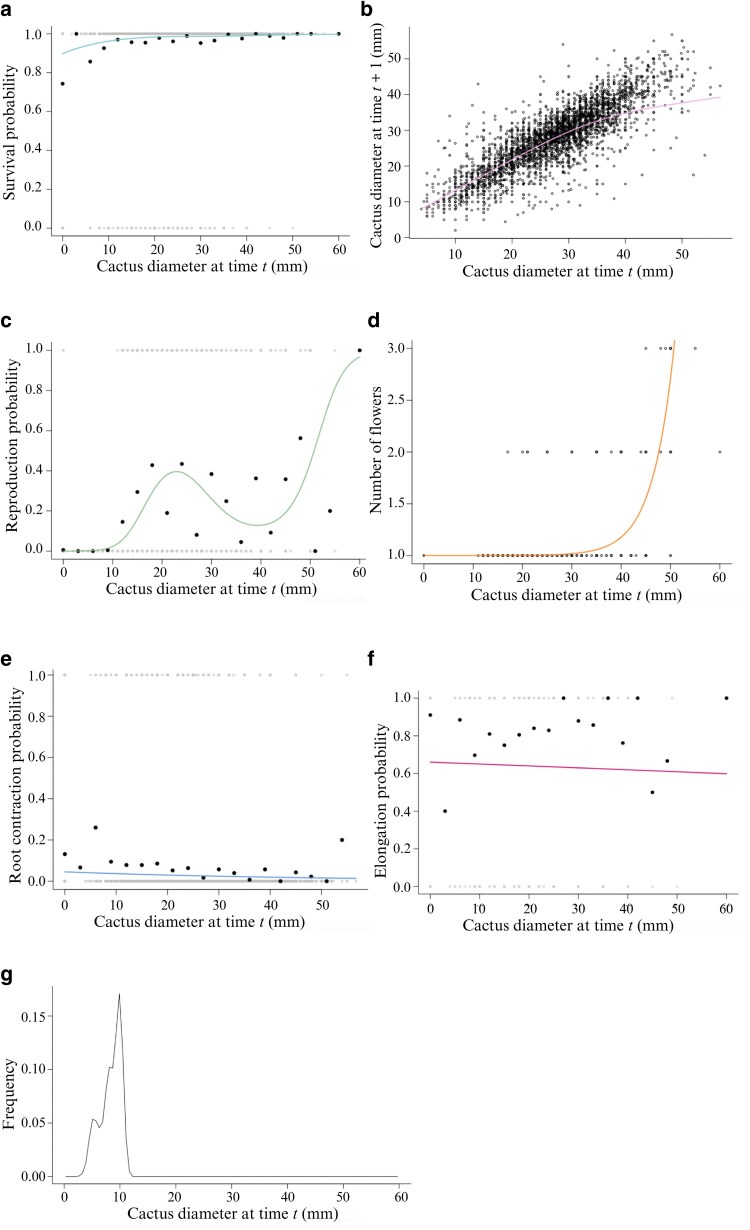
Vital rates models of *Pediocactus bradyi* as a function of individual size (diameter, mm). a) Survival probability, b) growth, c) reproduction probability, d) number of flowers produced by an individual, e) root contraction probability, f) elongation probability, and g) Offspring size distribution. Open circles correspond to individual measurements; closed circles in a), c), e), and f) correspond to averages over a 20-bin partition of the cactus size range.

The model that best fitted the survival data was non-linear, with all random effects except for the cactus on the intercept (Supplementary Table S1). These results indicate that the probability of survival is high from minimum diameter values and increases as the diameter of the individuals increases (Fig. 1a).

In the case of growth of individuals from time *t* to *t* + 1, the model that best fitted the data, according to the AIC, was non-linear (Supplementary Table S2). The fitted model indicates that individuals grow linearly and when they reach a certain size (∼35 mm in diameter), the growth rate decreases (Fig. 1b).

With respect to the probability of reproduction, the model that best fitted the data was non-linear (Supplementary Table S3). Starting at 10 mm in diameter, the probability of reproduction increases, reaching a high point at 25 mm in diameter (Fig. 1c); this probability then decreases and increases again at ∼40 mm, reaching a maximum point at the largest size values (60 mm).

For the number of reproductive structures, the model that best fitted the data was linear (Supplementary Table S4). The model shows that the number of reproductive structures per individual, in this case flowers, ranges from 1 to 3 (Fig. 1d). Most of the individuals with <30 mm in diameter generate only one flower; from 30 mm onwards, they begin to generate two or more flowers.

The probability of root contraction and the probability of elongation were modelled separately, with the best-fitted models being linear (Supplementary Tables S5 and S6), and the growth after elongation being non-linear (Supplementary Table S7). With respect to root contraction, the model indicates that this probability is low and decreases as size increases (Fig. 1e). Our data and the best-fitted model indicate that the probability of occurrence decreases as the diameter of the individuals increases. For the probability of elongation, we observe that it presents a higher probability with respect to contraction and this probability slightly decreases as the size of the individual increases (Fig. 1f).

Finally, the size structure of the offspring is mostly represented by individuals of 10 mm in diameter (Fig. 1g).

### Population dynamics analysis

**Figure 2:**
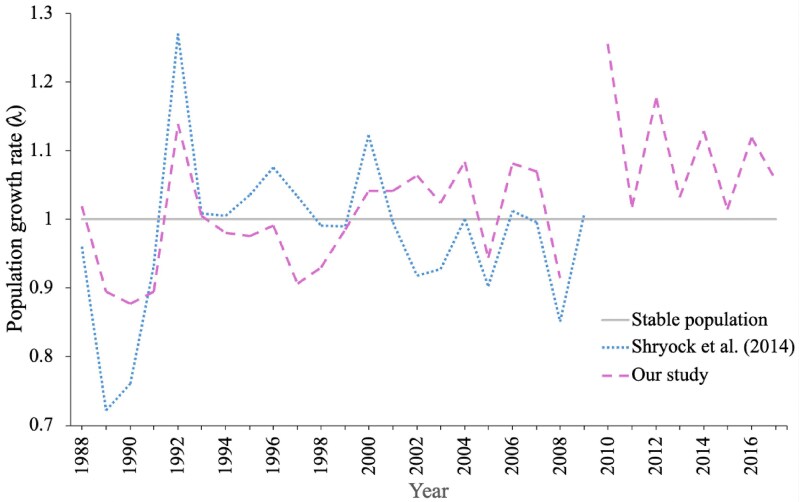
Estimated annual population growth rates of the *Pediocactus bradyi* population over the 1988–2017 study period. For comparison, the estimated rates obtained by Shryock et al. (2014) are also shown.

The average population growth rate over the study period was 1.022, which was higher than the one produced by Shryock et al. (2014) of 0.982. However, both studies reconstructed similar patterns (Fig. 2), with the worst years at the end of the 1980s followed by the best years in the first years of the 1990s, with our results being less fluctuating than those of Shryock et al. (2014). In more recent years, the population improved its performance as it remained over the one value threshold.

**Figure 3:**
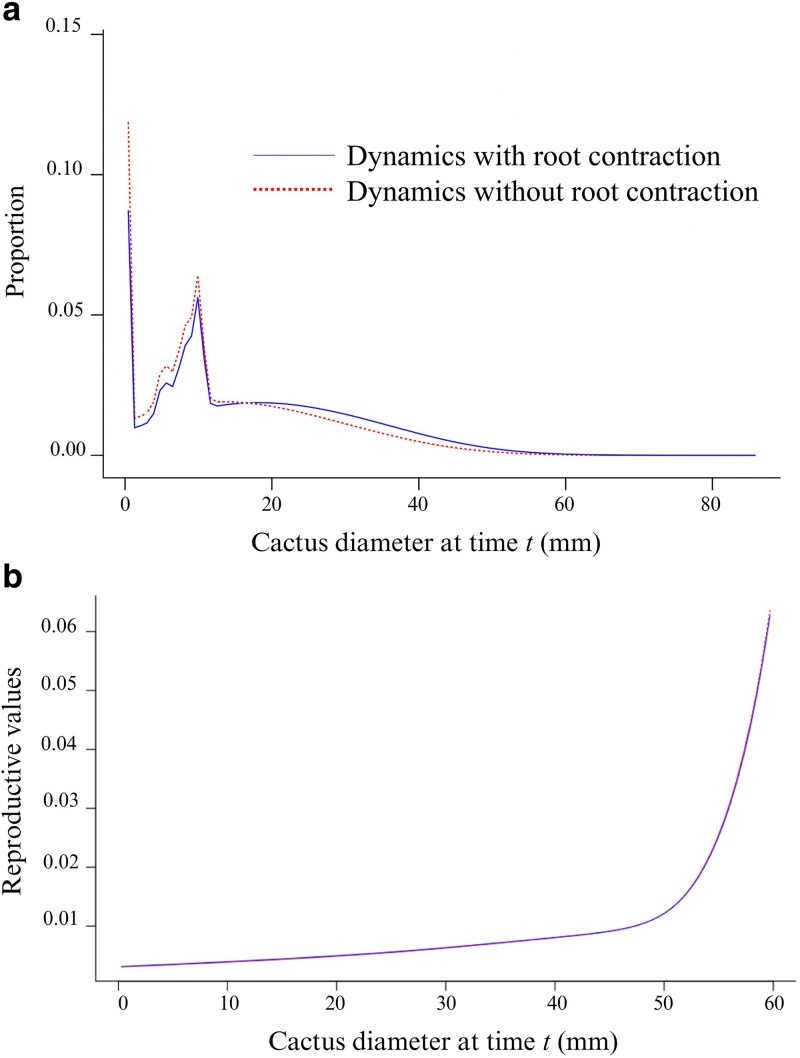
Stable size structure (a), and reproductive values (b) of the *Pediocactus bradyi* population average over the 1988–2017 study period. We show these population attributes as estimated from a population dynamics model that includes or excludes root contraction.

Comparing the models with and without root contraction, the value of the asymptotic population growth rate for the population dynamics without contraction was λ = 1.044, CI = (1.039, 1.303), while the rate with contraction was relatively similar, λ = 1.041, CI = (1.035, 1.289). The difference between these models is more evident at the level of the population structure, where the inclusion of root contraction translates into more individuals with larger sizes in comparison with the model with no contraction (Fig. 3a). In turn, the reproductive values are the same in both models, with larger individuals having the highest values (Fig. 3b).

Finally, the elasticity analysis indicated that a population dynamics that considers root contraction is largely affected by the survival of its individuals (*s*), followed by the growth in diameter of those individuals that show no contraction (*G_u_*) and the root elongation and growth of those individuals displaying elongation (*u* and *G_r_*, respectively) (Table 2).

**Table 2:** Elasticity values for each of the vital rates of the *Pediocactus bradyi* population analysed.

Vital rate	Elasticity
Survival (*s*)	0.39
Growth of non-root contracted individuals (*G_u_*)	0.24
Growth of root contracted individuals (*G_r_*)	0.15
Reproduction probability (*p_b_*)	0.02
Number of flowers (*b*)	0.02
Root contraction (*r*)	−0.01
Elongation (*u*)	0.17
Offspring size (*c*)	0.02

## Discussion

Our analysis covers 31 years of data and focuses on the survival, growth, reproduction, and root contraction in the *P. bradyi* population. We found that larger individuals showed higher survival and reproduction rates, likely due to their greater water storage capacity, while growth rates declined beyond 35 mm in diameter, possibly due to a reduced surface-to-volume ratio and unfavourable episodes of root contraction. Reproductive maturity was observed from 10 mm in diameter, earlier than previously reported by Hughes (2005). The probability of root contraction occurrence decreased with size, having little impact on overall population growth (λ). This last parameter indicated a stable population with a slight increase in recent years. Population structure was adult-biased, consistent with patterns seen in other long-lived cacti, and elasticity analyses showed that survival and growth, followed by root elongation after contraction, are the main vital rates affecting population growth.

### Vital rates

In general, vital rates were positively related to individual size. Thus, as the size of an individual increases, survival and reproductive values increase, with only root contraction probability decreasing with size. In particular, our analysis indicates a high probability of individual survival, which increases with plant diameter, likely because larger individuals can store more water, enhancing their resilience during prolonged drought periods (Gibson and Nobel 1986).

With respect to growth, the model that best fit the data was non-linear, with a decline in growth rates beginning around 35 mm in diameter. This is consistent with what Shryock et al. (2014) found and also consistent with other cacti species (Parker 1988, Félix-Burruel et al. 2019). In terms of the probability of reproduction and the number of reproductive structures, both were positively determined by plant size. Larger individuals tend to have more apical meristems, greater photosynthetic surface, and volume (Mauseth 2000, Williams et al. 2014), allowing them to fix more carbon and allocate additional resources to reproduction (Gibson and Nobel 1986). This trend is also observed in other globose cacti such as *Mammillaria magnimamma* and *M. supertexta* (Valverde et al. 2004, Avendaño 2007). Although Hughes (2005) reported the onset of flowering at ∼16 mm in diameter and defined adults from this threshold, our data show that some individuals as small as 10 mm had already produced at least one reproductive structure, i.e. they reach sexual maturity and can be considered adults.

Offspring sizes ranged in the 0–10 mm in diameter interval. The left skewness of this size distribution (Fig. 1g) may be due to a higher survival rate of the larger recruits, which gives them a higher probability of establishment. Another explanation could be associated with detectability, i.e. a sampling bias on the part of the observer in which smaller offspring can easily go unnoticed.

Regarding root contraction, our best-fit model suggests that its probability of occurrence decreases as individual diameter increases. This trend is likely due to the greater water storage capacity of larger individuals, which reduces their exposure to water stress. Hughes (2005) suggests that this phenomenon is an important factor in reducing mortality. In years with strong drought periods (1989, 2001, 2008), a large number of individuals were found to be retracted, and during years with very marked wet periods (1992, 1998, 2004), many of these retracted individuals surfaced. However, Shryock et al. (2014) state that the ability to retract below ground may not be enough to buffer against changes in the frequency or intensity of extreme climatic events. While an individual remains withdrawn, it does not grow, nor reproduce, and, therefore, does not contribute descendants to the population. This is why root elongation, which allows the individual to leave this state, has a positive impact on the overall population dynamics.

The results obtained in this work indicate that root contraction does not seem to affect population growth, since there is a significant overlap in the confidence intervals associated with λ. However, we are not considering climatic variables, which Hughes (2005) correlates with the occurrence of this phenomenon. According to Shryock et al. (2014), annual precipitation and higher winter minimum temperatures were correlated with population growth, with winter minimum temperature affecting λ but not mortality. Another example is the case of *Carnegiea gigantea*; in this species, temperature, and summer and winter drought affect reproduction and growth (Pierson et al. 2013, Hultine et al. 2018, Félix-Burruel et al. 2019), and precipitation affects recruitment (Félix-Burruel et al. 2021). For this reason, studies should be carried out that analyse the phenomenon of root contraction in relationship with climatic variables such as temperature, freezing, and precipitation.

Finally, we have to acknowledge that in this study, no retracted cactus was considered dead until it had remained in that state for >5 years; this decision may have affected the annual counts of individuals, increasing our uncertainty about annual population sizes (Bureau of Land Management 2005).

### Population dynamics and attributes

The average population growth rates (λ) that we obtained for both dynamics indicate that the population has been increasing over time: excluding root contraction, λ = 1.044, and including root contraction, λ = 1.041. For the same population, Shryock et al. (2014) obtained an average population growth rate of 0.982, which means that the population was decreasing according to their results. However, our study was conducted with 31 years of data, while Shryock et al. (2014) used 22 years. Other studies carried out for species of the genus *Mammillaria* have produced similar results, as in the case of *M. supertexta* whose λ was 1.088 (Avendaño 2007), for *M. huitzilopochtli* (Flores-Martínez and Manzanero 2005) a λ of 1.14 was obtained, and for *M. gaumeri* (Ferrer-Cervantes et al. 2012), year-specific λ values ranged from 0.83 to 1.48 during the period 1999–2008. In our case, year-specific λ values ranged in the 1.039–1.303 interval for the population dynamics that excluded root contraction, while the rate with root contraction was relatively similar, ranging from 1.035 to 1.289.

The stable size structure indicates that the *P. bradyi* population would be mostly composed of adult individuals if the population dynamics were maintained over time. This is in agreement with Godínez-Álvarez et al. (2003), who indicate that cacti populations are generally biased towards adults, due to the fact that recruitment is an infrequent phenomenon (Felix-Burruel et al. 2021). In contrast, Miller et al. (2012) indicate that, in the case of polycarpic plants, the population structure is generally dominated by juvenile individuals. Also, in the case of *P. bradyi*, the stable size structure agrees with the data observed in the field according to Hughes (2005), since the population of *P. bradyi* for the period from 1985 to 2004 was dominated by individuals between 20 and 30 mm in diameter, i.e. adults. Juvenile individuals between 0 and 15 mm in diameter were in the minority, except for the Badger Creek plot, which did show a dominance of juvenile individuals for the period 1994–9. Hughes (2005) mentions that individuals were considered adults from 16 mm in diameter because they were generating their first reproductive structures; however, our data indicate that this phenomenon is exhibited even in individuals as small as 10 mm in diameter (Fig. 1c), so they should be considered adults.

The reproductive values derived from both modelled dynamics (with and without root contraction) were very similar, differing by only 0.011 across the adjusted models (Fig. 3b). This consistency likely stems from reproduction occurring predominantly in non-retracted individuals. In all models, reproductive values increased with diameter, reflecting the greater capacity of larger individuals to generate reproductive structures and their higher likelihood of reproducing.

The elasticity analysis indicates that the vital rates that mostly affect λ are survival (*s*), the growth of non-retracted individuals (*G_u_*), root elongation (*u*), and the growth of elongated individuals (*G_r_*) (Table 2). The vital rates with the least impact on λ are those corresponding to fecundity (*p_b_*, *b*, and *c*) and root contraction (*r*), the latter affecting negatively. These results coincide with the study of Ferrer-Cervantes et al. (2012), in which their elasticity analysis reports that population dynamics strongly depend on survival; this pattern is common in long-lived cacti (Fernández-Muñiz et al. 2024, Jiménez-Guzmán et al. 2024).

Our results, although they coincide with the values reported in the literature for other globose cacti, have their limitations. We are not including climatic variables (Ferrer-Cervantes et al. 2012), nurse plants (Carrillo-Garcia et al. 1999), mycorrhizae (Rincón et al. 1993), or other factors that may positively or negatively affect the population growth rate. For example, the study by Shryock et al. (2014) mentions that the El Niño-driven precipitation pulses and high summer temperatures strongly impact seedling recruitment in the *P. bradyi* population we studied.

### Practical implications for conservation and management

Many conservation and management challenges are related to vital rates. To effectively protect endangered species, it is essential to enhance survival and fertility, thus promoting a balanced population growth and the reduction of the population extinction risk (Caswell 2001). For endangered species, understanding current population trends and projecting future scenarios are key strategies in guiding effective management (Martínez-Ramos 2016).

Elasticity analysis is fundamental to identify the relative relevance of vital rates for population growth, and thus for the design of conservation and management strategies (Jiménez-Guzmán et al. 2024). Our elasticity analysis found survival as the vital rate with the greatest influence on λ. Given that reproduction probability increases with individual size, that larger individuals are proportionally fewer, and that the species grows slowly, conservation efforts should prioritize enhancing large individual survival, irrespective of the root contraction status. These findings align with patterns observed in other cacti where recruitment is limited and growth is notably slow (Godínez-Álvarez et al. 2003, Ferrer-Cervantes et al. 2012).

Our population is located in the Navajo Nation, an area designated as a reserve since 1848. This protected status appears to have contributed positively to the species conservation, as the population did not decline during the study period. However, Shryock et al. (2014) did detect a decline in this population, and ascribed this decline mainly to herbivory through rodent predators (Hughes 2005). However, it is important to highlight that these contrasting results may be due to the larger number of years our study included. The reserve protection status must be preserved as it avoids human activities that could endanger population viability. Looting is a very common reason for population decline in this family due to their high value in the black market, specially in the largest size categories (Martínez-Ramos 2016). Nonetheless, given the species sensitivity to climate variation at recruitment, future changes in temperature and precipitation due to climate change could negatively impact the species long-term viability. Therefore, it is vital to develop adaptive conservation strategies that account for these potential environmental changes.

## Conclusions

Our results indicate that, although root contraction temporarily supports individual resilience, it appears to play a minimal impact on the population viability of the *P. bradyi* population; that individuals are capable of leaving this state through root elongation is in fact a more important process for population viability. As with many cacti species, survival was the most important vital rate for maintaining and promoting population growth, and thus stasis of larger individuals is fundamental to population resilience, as these individuals show higher survival and reproduction rates and are less susceptible to water stress and root contraction. Therefore, conservation efforts should focus on protecting adult individuals and mitigating threats such as poaching and the potential impacts of climate change.

## Supplementary Material

plaf074_Supplementary_Data

## Data Availability

Raw data and R code are available online at https://zenodo.org/records/17882366.
