# Supplementary material for: Root contraction does not increase long-term population growth in Pediocactus bradyi, an endangered cactus of Northern Arizona
Source: AoB Plants. 2025 Dec 30;18(1):plaf074. doi: 10.1093/aobpla/plaf074 (PMC12797314; doi:10.1093/aobpla/plaf074)
Supplement: plaf074_Supplementary_Data [file plaf074_supplementary_data.pdf]

Title: Root contraction does not increase long-term population growth in Pediocactus bradyi, an endangered cactus of Northern Arizona

Authors: Julieta Rojas-Pimentel, Eugenio Larios, Edgar J. González

Supporting Information. Tables of the models considered for the vital rates as a function of individual size.

Table S1. Models considered for survival probability as a function of size at time *t*. The selection criterion ΔAIC was used indicating the relative difference between the model with the best AIC and the other models considered; the abbreviation "NC" refers to models that did not converge; values 0 and 1 were used to indicate the absence or presence of random effects in model construction; the fixed effect refers to the variable of interest, in this case growth quantified in mm width. β_0 corresponds to the intercept of the linear regression and β_1 the slope.

	Random effects						
Fixed effect	Cactus		Year		Plot		ΔAIC
	β_0	β_1	β_0	β_1	β_0	β_1	
Null	0	0	0	0	0	0	597.1
Linear	1	1	1	1	1	1	7.1
Linear	0	1	1	1	1	1	3.4
Linear	0	1	1	0	1	1	3.9
Nonlinear	1	1	1	1	1	1	4

Nonlinear	0	1	1	1	1	1	0
Nonlinear	0	1	0	1	1	1	474.6

Table S2. Models considered for growth from time *t* to time *t* + 1. The selection criterion ΔAIC was used which indicates the relative difference between the model with the best AIC and the other models considered; the abbreviation NC indicates that the model does not converge; the values 0 and 1 were used to indicate the absence or presence of random effects in the construction of the model; the fixed effect refers to the type of model relating diameter at time *t* + 1 to diameter at time *t*. β_0 corresponds to the intercept of the linear regression and β_1 the slope.

Fixed effect	Random effects						ΔAIC
	Cactus		Year		Plot		
	β_0	β_1	β_0	β_1	β_0	β_1	
Null	0	0	0	0	0	0	2752.8
Linear	1	1	1	1	1	1	NC
Linear	1	1	1	1	1	0	NC
Linear	1	1	1	0	1	1	NC
Linear	1	0	1	1	1	1	NC
Linear	0	1	1	1	1	1	NC
Linear	1	1	0	1	1	1	NC
Linear	1	1	1	1	0	1	NC
Linear	0	0	1	1	1	1	NC
Linear	1	1	0	0	1	1	NC
Linear	1	1	1	1	0	0	NC

Linear	0	1	0	1	1	1	NC
Linear	1	1	0	1	0	1	NC
Linear	0	1	1	1	0	1	183.6
Linear	1	0	1	0	1	1	NC
Linear	1	1	1	0	1	0	NC
Linear	1	0	1	1	1	0	NC
Linear	0	1	1	0	1	1	NC
Linear	0	1	1	1	1	0	82.1
Linear	1	0	0	1	1	1	NC
Linear	1	1	0	1	1	0	NC
Linear	1	0	1	1	0	1	NC
Linear	1	1	1	0	0	1	NC
Linear	0	0	1	1	1	0	126.7
Nonlinear	1	1	1	1	1	1	0
Nonlinear	1	1	1	1	1	0	21.4

Table S3. Models considered for the probability of reproduction as a function of size at time *t*. The selection criterion ΔAIC was used indicating the relative difference between the model with the best AIC and the other models considered; the abbreviation "NC" refers to models that did not converge; values 0 and 1 were used to indicate the absence or presence of random effects in model construction; the fixed effect refers to the variable of interest, in this case growth quantified in mm width. β_0 corresponds to the intercept of the linear regression and β_1 the slope.

Fixed effect	Random effects						ΔAIC
	Cactus		Year		Plot		
	β_0	β_1	β_0	β_1	β_0	β_1	
Null	0	0	0	0	0	0	1899
Linear	1	1	1	1	1	1	NC
Linear	0	1	1	1	1	1	NC
Linear	1	1	0	1	1	1	NC
Linear	1	1	1	1	0	1	NC
Linear	1	0	1	1	1	1	NC
Linear	1	1	1	0	1	1	NC
Linear	1	1	1	1	1	0	NC
Linear	0	0	1	1	1	1	NC
Linear	1	1	0	0	1	1	NC
Linear	1	1	1	1	0	0	NC

Linear	0	1	0	1	1	1	NC
Linear	1	1	0	1	0	1	NC
Linear	0	1	1	1	0	1	NC
Linear	1	0	1	0	1	1	NC
Linear	1	1	1	0	1	0	431
Linear	1	0	1	1	1	0	NC
Linear	0	1	1	0	1	1	NC
Linear	0	1	1	1	1	0	NC
Linear	1	0	0	1	1	1	NC
Linear	1	1	0	1	1	0	NC
Linear	1	0	1	1	0	1	NC
Linear	1	1	1	0	0	1	NC
Linear	1	0	1	0	1	0	432.4
Nonlinear	1	1	1	1	1	1	7.6
Nonlinear	0	1	1	1	1	1	2
Nonlinear	0	0	1	1	1	1	0
Nonlinear	0	0	1	1	0	1	35.6

Table S4. Models considered for the number of reproductive structures as a function of size at time *t*. The selection criterion ΔAIC was used indicating the relative difference between the model with the best AIC and the other models considered; the abbreviation "NC" refers to models that did not converge; values 0 and 1 were used to indicate the absence or presence of random effects in model construction; the fixed effect refers to the variable of interest, in this case growth quantified in mm width. β_0 corresponds to the intercept of the linear regression and β_1 the slope.

	Random effects						
Fixed effect	Cactus		Year		Plot		ΔAIC
	β_0	β_1	β_0	β_1	β_0	β_1	
Null	0	0	0	0	0	0	860.5
Linear	1	1	1	1	1	1	NC
Linear	0	1	1	1	1	1	NC
Linear	1	1	0	1	1	1	NC
Linear	1	1	1	1	0	1	NC
Linear	1	0	1	1	1	1	NC
Linear	1	1	1	0	1	1	NC
Linear	1	1	1	1	1	0	NC
Linear	0	0	1	1	1	1	NC
Linear	1	1	0	0	1	1	NC
Linear	1	1	1	1	0	0	NC

Linear	0	1	0	1	1	1	NC
Linear	1	1	0	1	0	1	NC
Linear	0	1	1	1	0	1	NC
Linear	1	0	1	0	1	1	NC
Linear	1	1	1	0	1	0	NC
Linear	1	0	1	1	1	0	NC
Linear	0	1	1	0	1	1	NC
Linear	0	1	1	1	1	0	NC
Linear	1	0	0	1	1	1	NC
Linear	1	1	0	1	1	0	NC
Linear	1	0	1	1	0	1	NC
Linear	1	1	1	0	0	1	NC
Linear	0	1	0	1	0	1	10.5
Linear	1	0	1	0	1	0	10.5
Linear	0	0	0	1	1	1	NC
Linear	0	0	1	1	0	1	NC
Linear	0	0	1	0	1	1	NC
Linear	0	0	1	1	1	0	NC
Linear	0	1	0	0	1	1	NC

Linear	1	1	0	0	0	1	NC
Linear	1	0	0	0	1	1	NC
Linear	1	1	0	0	1	0	NC
Linear	0	1	1	1	0	0	NC
Linear	1	1	0	1	0	0	NC
Linear	1	0	1	1	0	0	NC
Linear	1	1	1	0	0	0	NC
Linear	0	1	0	1	1	0	1.8
Linear	1	0	0	1	0	1	NC
Linear	0	1	1	0	0	1	NC
Linear	1	0	1	0	0	1	NC
Linear	0	1	1	0	1	0	NC
Linear	1	0	0	1	1	0	NC
Linear	0	0	0	1	1	0	0
Linear	0	0	0	0	1	0	1.4
Nonlinear	1	1	1	1	1	1	7.8
Nonlinear	1	1	1	1	1	0	3.9
Nonlinear	1	1	1	0	1	0	9

Table S5. Models considered for root contraction probability as a function of size at time *t*. The selection criterion ΔAIC was used indicating the relative difference between the model with the best AIC and the other models considered; the abbreviation “NC” refers to models that did not converge; values 0 and 1 were used to indicate the absence or presence of random effects in model construction; the fixed effect refers to the variable of interest, in this case growth quantified in mm width. β_0 corresponds to the intercept of the linear regression and β_1 the slope.

	Random effects						
Fixed effect	Cactus		Year		Plot		ΔAIC
	β_0	β_1	β_0	β_1	β_0	β_1	
Null	0	0	0	0	0	0	644.2
Linear	1	1	1	1	1	1	NC
Linear	0	1	1	1	1	1	NC
Linear	1	1	0	1	1	1	NC
Linear	1	1	1	1	0	1	NC
Linear	1	0	1	1	1	1	NC
Linear	1	1	1	0	1	1	NC
Linear	1	1	1	1	1	0	NC
Linear	0	0	1	1	1	1	NC
Linear	1	1	0	0	1	1	NC
Linear	1	1	1	1	0	0	NC

Linear	0	1	0	1	1	1	NC
Linear	1	1	0	1	0	1	NC
Linear	0	1	1	1	0	1	NC
Linear	1	0	1	0	1	1	0
Linear	1	1	1	0	1	0	NC
Linear	1	0	1	1	1	0	4.3
Linear	0	1	1	0	1	1	NC
Linear	0	1	1	1	1	0	NC
Linear	1	0	0	1	1	1	NC
Linear	1	1	0	1	1	0	NC
Linear	1	0	1	1	0	1	NC
Linear	1	1	1	0	0	1	NC
Linear	1	0	1	0	1	0	1
Nonlinear	1	1	1	1	1	1	9
Nonlinear	1	0	1	1	1	1	5.4
Nonlinear	1	0	1	0	1	1	2
Nonlinear	1	0	1	0	1	0	3

Table S6. Models considered for the probability of elongation as a function of size at time *t*. The selection criterion ΔAIC was used indicating the relative difference between the model with the best AIC and the other models considered; the abbreviation "NC" refers to models that did not converge; values 0 and 1 were used to indicate the absence or presence of random effects in model construction; the fixed effect refers to the variable of interest, in this case growth quantified in mm width. β_0 corresponds to the intercept of the linear regression and β_1 the slope.

Fixed effect	Random effects						ΔAIC
	Cactus		Year		Plot		
	β_0	β_1	β_0	β_1	β_0	β_1	
Null	0	0	0	0	0	0	102.7
Linear	1	1	1	1	1	1	NC
Linear	0	1	1	1	1	1	NC
Linear	1	1	0	1	1	1	NC
Linear	1	1	1	1	0	1	NC
Linear	1	0	1	1	1	1	NC
Linear	1	1	1	0	1	1	NC
Linear	1	1	1	1	1	0	NC
Linear	0	0	1	1	1	1	NC
Linear	1	1	0	0	1	1	NC

Linear	1	1	1	1	0	0	NC
Linear	0	1	0	1	1	1	NC
Linear	1	1	0	1	0	1	NC
Linear	0	1	1	1	0	1	NC
Linear	1	0	1	0	1	1	NC
Linear	1	1	1	0	1	0	NC
Linear	1	0	1	1	1	0	7.9
Linear	0	1	1	0	1	1	NC
Linear	0	1	1	1	1	0	NC
Linear	1	0	0	1	1	1	NC
Linear	1	1	0	1	1	0	NC
Linear	1	0	1	1	0	1	NC
Linear	1	1	1	0	0	1	NC
Linear	0	0	1	1	1	0	5.9
Linear	0	0	1	1	0	0	3.9
Linear	0	0	1	0	0	0	0
Nonlinear	1	1	1	1	1	1	NC
Nonlinear	0	1	1	1	1	1	13.5
Nonlinear	1	1	0	1	1	1	NC

Nonlinear	1	1	1	1	0	1	NC
Nonlinear	1	0	1	1	1	1	13.5
Nonlinear	1	1	1	0	1	1	NC
Nonlinear	1	1	1	1	1	0	NC
Nonlinear	0	0	1	1	1	1	11.5
Nonlinear	0	0	1	1	1	0	7.9
Nonlinear	0	0	1	0	1	0	4
Nonlinear	0	0	1	0	0	0	2

Table S7. The selection criterion ΔAIC indicating the relative difference between the model with the best AIC and the other models considered was used; the abbreviation "NC" refers to models that did not converge; values 0 and 1 were used to indicate the absence or presence of random effects in model construction; the fixed effect refers to the variable of interest, in this case growth quantified in mm width. β_0 corresponds to the intercept of the linear regression and β_1 the slope.

Fixed effect	Random effects						ΔAIC
	Cactus		Year		Plot		
	β_0	β_1	β_0	β_1	β_0	β_1	
Null	0	0	0	0	0	0	2804.5
Linear	1	1	1	1	1	1	NC
Linear	1	1	1	1	1	0	NC
Linear	1	1	1	0	1	1	NC
Linear	1	0	1	1	1	1	NC
Linear	0	1	1	1	1	1	NC
Linear	1	1	0	1	1	1	NC
Linear	1	1	1	1	0	1	NC
Linear	0	0	1	1	1	1	NC
Linear	1	1	0	0	1	1	NC
Linear	1	1	1	1	0	0	NC

Linear	0	1	0	1	1	1	NC
Linear	1	1	0	1	0	1	NC
Linear	0	1	1	1	0	1	191
Linear	1	0	1	0	1	1	NC
Linear	1	1	1	0	1	0	NC
Linear	1	0	1	1	1	0	NC
Linear	0	1	1	0	1	1	NC
Linear	0	1	1	1	1	0	82.1
Linear	1	0	0	1	1	1	NC
Linear	1	1	0	1	1	0	NC
Linear	1	0	1	1	0	1	NC
Linear	1	1	1	0	0	1	NC
Linear	0	1	1	1	0	0	189.02
Linear	0	0	1	1	0	0	229.8
Nonlinear	1	1	1	1	1	1	0
Nonlinear	1	1	1	1	1	0	24.67
